# Mitigating Health Risks to Reopen a Clinical Research Laboratory During the COVID-19 Pandemic: A Framework

**DOI:** 10.2196/22570

**Published:** 2020-12-04

**Authors:** Eloise M Walker, Alice E Jasper, Lauren Davis, Kay Por Yip, Aduragbemi A Faniyi, Michael J Hughes, Helena A Crisford, Daniella A Spittle, Elizabeth Sapey, Kylie B R Belchamber, Aaron Scott

**Affiliations:** 1 Birmingham Acute Care Research Group Institute of Inflammation and Ageing University of Birmingham Birmingham United Kingdom; 2 NIHR Clinical Research Facility Birmingham University Hospitals Birmingham NHS Foundation Trust Birmingham United Kingdom

**Keywords:** clinical laboratory, risk assessment, COVID-19, SARS-CoV-2, framework, research, risk, lab, safety

## Abstract

**Background:**

The COVID-19 pandemic has led to many countries implementing lockdown procedures, resulting in the suspension of laboratory research. With lockdown measures now easing in some areas, many laboratories are preparing to reopen. This is particularly challenging for clinical research laboratories due to the dual risk of patient samples carrying the virus that causes COVID-19, SARS-CoV-2, and the risk to patients being exposed to research staff during clinical sampling. To date, no confirmed transmission of the virus has been confirmed within a laboratory setting; however, operating processes and procedures should be adapted to ensure safe working of samples of positive, negative, or unknown COVID-19 status.

**Objective:**

In this paper, we propose a framework for reopening a clinical research laboratory and resuming operations with the aim to maximize research capacity while minimizing the risk to research participants and staff.

**Methods:**

This framework was developed by consensus among experienced laboratory staff who have prepared to reopen a clinical research laboratory.

**Results:**

Multiple aspects need to be considered to reopen a clinical laboratory. We describe our process to stratify projects by risk, including assessment of donor risk and COVID-19 clinical status, the COVID-19 status of the specific sample type, and how to safely process each sample type. We describe methods to prepare the laboratory for safe working including maintaining social distancing through signage, one-way systems and access arrangements for staff and patients, limiting staff numbers on site and encouraging home working for all nonlaboratory tasks including data analysis and writing. Shared equipment usage was made safe by adapting booking systems to allow for the deployment of cleaning protocols. All risk assessments and standard operating procedures were rewritten and approved by local committees, and staff training was initiated to ensure compliance.

**Conclusions:**

Laboratories can adopt and adapt this framework to expedite reopening a clinical laboratory during the current COVID-19 pandemic while mitigating the risk to research participants and staff.

## Introduction

The novel coronavirus SARS-CoV-2, causing the disease COVID-19, emerged from China in December 2019 with the World Health Organization declaring the outbreak a public health emergency of international concern on January 30, 2020. Since then, many countries have implemented lockdown measures with only essential work allowed. This has impacted many workplaces, including laboratories, which have had to suspend research. With lockdown measures being gradually eased, many laboratories are now preparing to reopen. Reopening clinical research laboratories in the wake of COVID-19 presents a particular challenge because many patients’ biological samples are likely to have an unknown SARS-CoV-2 status at the time of sampling. The Advisory Committee on Dangerous Pathogens have classified SARS-CoV-2 as a HG3 (Hazard Group 3) pathogen [1]. This requires specific procedures to be implemented under stringent safety approval processes. Many laboratories will not meet the specific requirements needed to handle and process samples that are COVID-19 positive or have unknown status. The transmission risks and pathogenicity of this virus is still unclear. However, there is growing scientific evidence on many aspects of the virus biology, and this has helped to inform public health measures including the reopening of workplaces.

SARS-CoV-2 is capable of human to human transmission, with aerosol transmission of the virus also documented [2-4]. The virus is mainly spread through inhalation of respiratory droplets produced when an infected individual coughs or sneezes [5-7]. Respiratory droplets can also contaminate surfaces, and as such, fomite transmission can occur. Current evidence indicates that the virus may survive up to 72 hours on stainless steel and plastic with a half-life of approximately 5.6 hours and up to 24 hours on cardboard and up to 4 hours on copper [4]. However, whether the virus is still capable of causing infection over this time course has yet to be demonstrated. The half-life of SARS-CoV-2 in aerosols was shown to be approximately 1.2 hours [4]. To prevent spread, current government guidelines specify a social distance from persons not from the same household of 1-2 meters and encourage regular hand washing for at least 20 seconds in soapy water [8].

The clinical symptoms of COVID-19 are varied, ranging from no symptoms (asymptomatic) and those mimicking a common cold to a severe respiratory disease including pneumonia and progression to acute respiratory distress syndrome. Early symptoms of COVID-19 are characterized by one or more of the following: a persistent cough, a fever, and a change in or loss of taste and/or smell [9,10]. Current government guidelines state that if one of more of these symptoms is experienced, a person must self-isolate for 14 days. Evidence suggests a temporal pattern of viral shedding with viral load at its highest shortly after disease onset with a gradual decrease over time [11,12].

To date, the evidence on the role of asymptomatic carriers in SARS-CoV-2 transmission is unclear. However, the virus has been detected in individuals who do not exhibit any symptoms of the disease [13]. Until more is known, it is important to consider the possible risk of asymptomatic carriage when assessing the risks to individuals within a workplace. In the laboratory setting, it may not be possible to test patients prior to biological sample donation.

Levels of viral RNA (ribonucleic acid) detected in blood samples appear to be low [14]. In a study carried out on 307 blood samples from patients with COVID-19, only 1% tested positive via polymerase chain reaction (PCR). No viral RNA was detected in urine samples from the same patients [15]. These sample types therefore pose a lower risk to individuals handling them compared to respiratory tract samples. Bronchoalveolar lavage fluid specimens and sputum samples showed the highest viral loads detected with 93% and 72% of samples with a positive PCR result, respectively [15]. Upper respiratory tract samples, nasal swabs, and pharyngeal swabs have been routinely used for testing during the COVID-19 outbreak. A study comparing viral detection in nasal swabs and saliva samples in persons not displaying any obvious clinical signs of COVID-19 found that not only was the virus detected in saliva samples, but that the cycle threshold (Ct) values were lower than that of throat swabs from the same individuals, suggesting that viral load may be higher in saliva than in throat swab samples. Individuals testing positive by saliva samples later went on to develop clinical symptoms [16].

To date, no confirmed transmission of the virus has been confirmed within a laboratory setting. However, laboratory work must be altered, and the appropriate processes and procedures need to be put in place to ensure a safe working environment if samples are of positive or unknown COVID-19 status.

SARS-CoV-2 is easily destroyed by alcohol [17] and soap [18] due to disruption of the protein envelope. Therefore, handwashing remains an extremely effective way of breaking fomite transmission; 70% ethanol is an effective disinfectant within a laboratory setting.

There remain considerable uncertainties in reopening clinical research laboratories that process human samples and have a disease-based focus. Resuming laboratory activities including sample taking and processing requires consideration of a number of risks, which, broadly speaking, can be divided into:

- Risks to the research participant (in attending the clinical laboratory setting and being close to research staff or other research participants)
- Risks to the research staff (from exposure to patients, processing samples, and working with others in a laboratory facility)

This paper provides a suggested framework that may inform how clinical laboratory operations can be resumed with the aim to maximize research capacity while minimizing the risk to research participants and staff.

## Methods

This framework was developed by consensus among experienced laboratory staff who have prepared to reopen a clinical research laboratory. Staff included 1 consultant in respiratory medicine and professor of acute and respiratory medicine, 1 respiratory registrar, and 8 immunologists working in a translational laboratory.

## Results

### Project Classification and Risk Assessment

To allow resumption of research as quickly but safely as possible, studies/projects should be stratified into categories by risk. This includes the participants’ demographics, medical history (with relevant bodies providing definitions of the levels of risk depending on disease area and severity [19,20]) and the likely COVID-19 status of the sample (positive, negative, or unknown). Ethically approved protocols may require amendments to incorporate these factors to mitigate risk where possible. It is also important to consider the status of the building that participants will be entering (hot, cold, or mixed site), and their ingress and egress routes through the facility. We recommend a risk assessment for every participant, which includes consideration of their medical history, likelihood of having COVID-19 (based on screening questionnaires), and the samples to be collected.

#### Assessment of Donor Risk and COVID-19 Clinical Status

Clinical research often requires samples from individuals presenting with existing medical conditions, which may predispose them to a more severe outcome if infected with SARS-CoV-2 [21-23]. These include demographic factors such as age [23,24], ethnicity [18], and smoking status [24-26]. It is important to identify which potential donors are high risk prior to attending clinical or research spaces in accordance with current government guidelines and initially prioritizing research participation for those deemed at less risk, if possible.

Some participants will have a COVID-19 swab taken as part of their routine care (such as those admitted to hospitals or where directed by clinical care pathways). Where the participant is known to have COVID-19 infection, the risk to staff and the participant from donating samples are known and can be mitigated with appropriate personal protective equipment (PPE) and HG3 laboratory processing. Where COVID-19 is highly suspected clinically but not confirmed by SARS-CoV-2 swab, we recommend treating the sample as though positive, as per national guidelines.

A negative swab cannot fully exclude COVID-19 infection due to the false-negative rates, but when combined with a low clinical suspicion of COVID-19 infection, could be assessed as “COVID-19 unlikely” in status, although many laboratories would still suggest using enhanced category 2 (Cat2+) for processing.

In some instances, the COVID-19 status of the participant will be unknown (with no swab taken). Screening questionnaires to identify and avoid participants with potential COVID-19–related symptoms may help mitigate risk further, and following that, if clinical suspicion is low, Cat2+ processing should still be considered.

**Figure 1:**
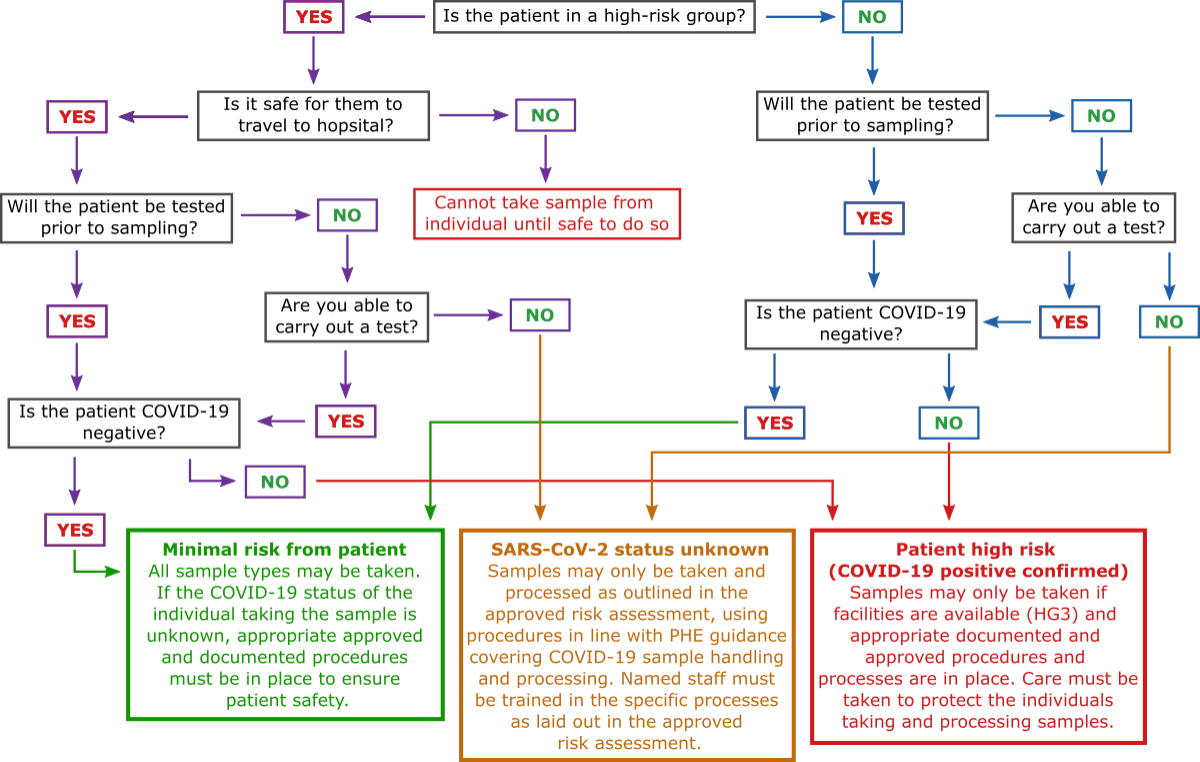
Donor risk flow diagram. Patients are first assessed by phone by a health care professional in the form of a questionnaire. If the patient is unable to be tested for COVID-19, the sample must be processed as status unknown according to Public Health England (PHE) guidance. If the patient can be tested and the test is returned negative, it is safe to assume minimal risk and proceed according to local risk assessments. If the test is returned positive, samples can only be taken if Hazard Group 3 (HG3) facilities are available and proceed according to local risk assessments.

We propose a simple workflow (Figure 1) to aid in stratifying donor groups by risk and capacity of the laboratory for handling high-risk pathogens.

In brief, research donors may be screened remotely by an appropriately trained health care professional prior to presenting at the clinic to determine medical conditions, which may increase risk and active symptoms or known exposure to COVID-19. An example of this is presented in Multimedia Appendix 1. Once the health care professional is reassured that the individual is not an obvious risk, they may be invited to attend the clinic. Upon arrival, the individual should be reassessed to identify characteristic COVID-19 symptoms and signs such as an elevated temperature. If resources allow, individuals presenting both with or without clinical symptoms may be tested using an approved and recommended test such as PCR to confirm COVID-19 status, in the understanding that the time between swab and result may necessitate multiple patient visits to the research department. In the event that a test is not available or feasible, samples from patients with low clinical suspicion of COVID-19 may be taken for research. However, the appropriate PPE and procedures must be followed when taking samples which then must still be treated with caution during processing as asymptomatic carriage is common [13].

#### COVID-19 Status of Sample and Risk Assessment

**Figure 2:**
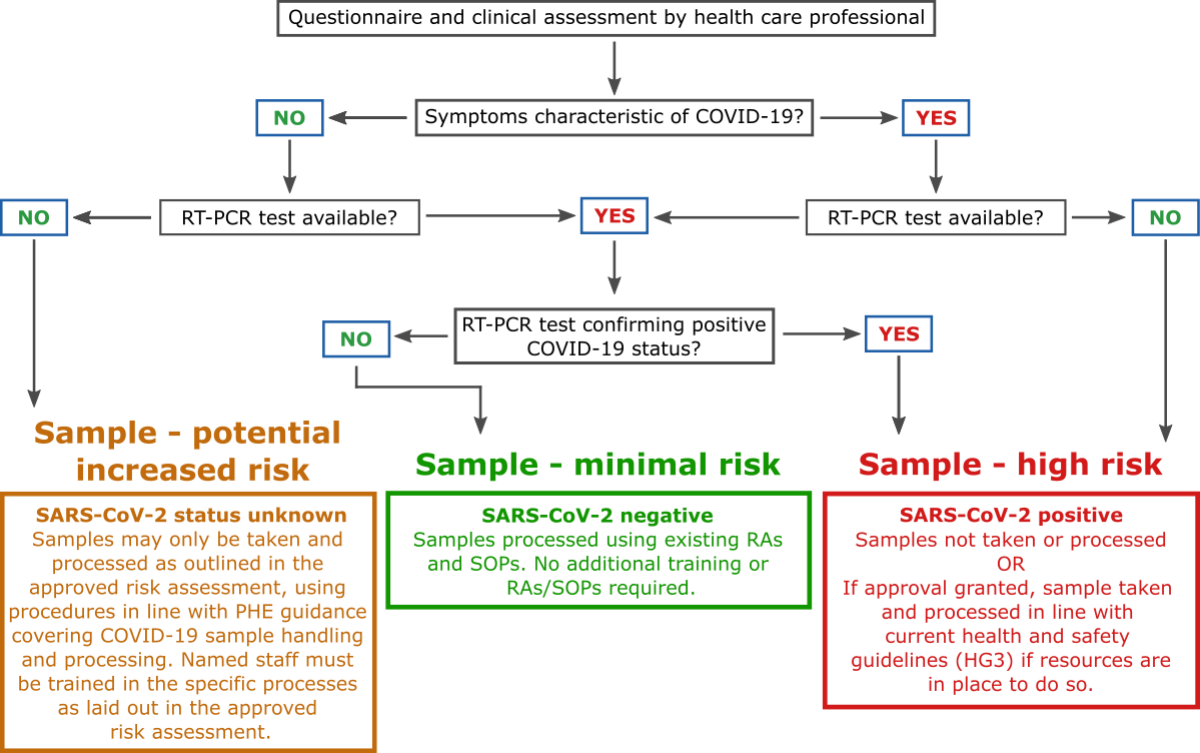
Clinical laboratory flow diagram for assessing sample risk. Individuals are first assessed by phone by a health care professional in the form of a questionnaire. If no clear risk is identified, patients may present at a clinic. Here, they will undergo a clinical observation to assess for characteristic symptoms of COVID-19. If resources allow, they may then undergo testing for the virus. The information will identify the risk of a particular patient sample and will inform decisions as to which samples can be taken and which can be processed safely. HG3: Hazard Group 3; PHE: Public Health England; RA: risk assessment; RT-PCR: reverse transcription-polymerase chain reaction; SOP: standard operating procedure.

SARS-CoV-2 has been detected in several different clinical sample types, including blood, feces, and respiratory specimens (Table 1). Risk assessments for taking samples such as phlebotomy should be reviewed, amended, and approved before study resumption. Assessing the risk of each sample is imperative, and we propose a tricolor risk alert system as demonstrated in the flow diagram in Figure 2.

**Table 1:** Viral detection and cultivation of live virus in clinical samples to date.

Sample type	Virus detected by PCR^a^	Live virus cultivated	Reference
Nasal swab	Yes	Yes	Yang et al [27]; Wang et al [15]
Throat swab	Yes	Yes	Yang et al [27]; Wang et al [15]
Saliva	Yes	Yes	To et al [11]
Sputum	Yes	Yes	Yang et al [27]; Wang et al [15]
Bronchoalveolar lavage	Yes	Yes	Yang et al [27]; Wang et al [15]
Lung tissue	Yes	Yes	Wang et al [15]
Feces and rectal swab	Yes	No	Wang et al [15]; To et al [11]
Urine	Yes	No	To et al [11]
Blood	Yes	No	To et al [11]; Wang et al [15]
Sperm	Yes	No	Li et al [28]

^a^PCR: polymerase chain reaction.

All samples with an unknown COVID-19 status (even if the risk is assessed as low; eg, whole blood sample from donor who has no clinical symptoms) should be processed under Cat2+ conditions, including use of a class II or class I microbiological safety cabinet in a category 2 laboratory but under aerosol tight conditions. Such procedures are specified in the risk assessment example in the online supplement. Any samples taken out of the cabinet should be placed inside at least two levels of containment. Samples used for processes performed outside of a microbiological safety cabinet such as flow cytometry or RNA extraction should first be inactivated or fixed by an approved and tested method such as 4% paraformaldehyde [25]. This should be documented in standard operating procedures and risk assessments. Any procedures involving the possible production of aerosols such as pipetting, vortexing, flow cytometry, and centrifugation should be risk assessed and the appropriate, approved procedures implemented. Samples may only be processed if the infrastructure and experience of staff allows for it.

Cells from samples of unknown COVID-19 status must not be put into culture as this may inadvertently result in amplification of the virus if it is present [29-31].

#### High-Risk Patients and Samples

Any sample from an individual with confirmed COVID-19 must be classified as high risk and treated as such. The highest viral loads have been detected in respiratory specimens [15], and as such these samples are deemed high risk if the COVID-19 status of the patient is positive or unknown. This includes bronchoalveolar lavage, sputum, and lung tissue samples [31], and therefore we recommend only processing lung tissue if the patient has been screened for COVID-19 and has been confirmed negative or low risk. All high-risk samples must be processed at category 3 level using appropriate containment. If such containment or trained staff are not available, such samples should not be taken or processed. New risk assessments should be written and official safety approval granted before working with high-risk specimens.

#### High-Risk Patients and Lower-Risk Samples

Viral presence in blood and urine has been shown to be varied; however, current evidence suggests that the virus is not always detectable in the blood or urine of COVID-19–confirmed cases. When it is, levels are low with no current evidence of infectivity from these samples. These sample types are therefore at a lower risk than respiratory samples. These samples can be processed within the context of amended risk assessments and official safety approval and in line with current safety guidelines, which will inform laboratories of the containment level required. Under current guidelines, this mandates Cat2+ conditions, operating under aerosol-free conditions at all times.

#### Low-Risk Patients

Samples taken from individuals with a confirmed COVID-19 negative test result are deemed to pose no added risk. As such, these samples can be processed and handled under the standard laboratory operating procedures with risk assessments and safety approval already in place. No further action is needed beyond the requirement to make the workplace safe as indicated below. Where the status of a patient is unknown, but clinical suspicion is low, we recommend processing within Cat2+ conditions, operating under aerosol-free conditions at all times.

### Preparing the Laboratory for Safe Working

Social distancing is vital in a pandemic as discussed in a recent systematic review and meta-analysis by Chu et al [32], where a physical distance of 1 meter reduced the risk of betacoronavirus transmission by 82% and that every additional 1-meter increase in social distance doubled the relative protection. To prevent the risk of transmission between persons and through fomites, adherence to current government social distancing guidelines is necessary. Outlined below are several actionable suggestions to increase the safety of the workforce and to help staff to maintain distancing while maximizing research capacity.

### Signage, One-Way Systems, and Access Arrangements

In order to maintain social distance within the laboratory/workplace, clear rules and signage should be put into place as follows:

- Clear signage and demarcations: signs on doors to laboratories, equipment rooms, offices, and toilets to indicate the number of persons inside at any given time. This will ensure that the capacity of the room is not exceeded and will allow persons to only enter the room if the capacity has not already been met. Signage may also indicate which specific working groups occupy a room at any given time. This can be in the form of a magnetic board or white board.
- A knock and call system: a knock and call procedure may be adopted for entering certain areas such as toilet areas.
- One-way systems around the building: if the layout of the workplace allows for this, one-way systems with clear signage will prevent individuals passing each other and will enable staff to maintain social distance while moving around the building. If the layout of the building does not allow this, the corridors may be split in two with directional traffic on each side. Directional systems must be clearly marked.
- Restricted access: it may be desirable to limit access to only specifically trained staff members to be on site. This will ensure that only authorized persons may gain access to the site. Staff should have their staff ID exhibited clearly at all times.

### Identifying High-Risk Staff, Limiting Staff Numbers on Site, and Encouraging Working From Home

In order to reduce the risk to staff of catching and spreading the virus, clear rules must be put in place as follows:

- Identifying high-risk staff: there is clear evidence that certain groups of people are at a higher risk of severe illness if they contract COVID-19. In order to protect staff, it is essential to identify any individual who may fall into a high-risk category such as pregnant women or individuals with underlying medical conditions. Prior to returning to work, such individuals must have a meeting with their line manager to discuss potential hazards and work options. They must follow the current government recommendations on shielding of high-risk groups. This may mean that such staff members are temporarily moved onto other projects that only require working from home until it is safe for them to return to the laboratory. Alternatively, if space allows, designated work areas may be provided to avoid contact with other staff members.
- Encouraging working from home: unlike many other workplaces, laboratory work cannot be done from home. However, work such as data analysis, experiment planning, stock ordering, and writing should be done at home where possible. In the event that data analysis requires a specific software, piece of equipment, or there is limited access due to data security, procedures must be followed to make staff members safe as outlined below.
- Limiting the number of staff on site: in order to maintain social distancing, numbers of staff members on site will need to be reduced. This can be done by a combination of facilitating working from home where possible, by implementing a rota system and by prioritizing projects.

### Work Pods, Rotas, and Shared Working Strategies

In order to meet social distancing guidelines, clear rules must be put in place as follows:

- Creating working groups (pods): restricting work activity to only occur within a small group of staff will limit spread amongst workers if an outbreak in the workplace does occur. Where possible, staff should be split into pods, whereby only members of each pod can occupy the laboratory or work space together at one time. When pods switch, all common areas must be cleaned. In the event that one member of a pod becomes ill and displays characteristic symptoms of COVID-19, all members of the pod must self-isolate until they have had a test confirming that they do not harbor the virus. Only then may they return to work. Splitting staff members into pods means that if one pod is required to isolate, other pods may continue to work. This helps to maintain research capacity and protects workers.
- Implementing work rotas and shift patterns: in order to meet social distancing rules and to maintain work pods, rotas will need to be established. Frequently changing requirements are common in translational research. As such, work rotas will need constant management to meet as many requirements of staff members as possible without breaking the pod system. We have found a daily rota split into two sessions (8:15 AM to 1 PM and 1:15 PM to 6 PM) is the best option for maximum flexibility. A short time period in between pod switchover will ensure that individual pods do not come into contact with each other when entering and exiting the building. On some days, a pod may take up both sessions. Time must be allocated at the end of each shift to allow for cleaning of the work area to prepare for the arrival of the next pod.
- Teamwork and work sharing: teamwork will be essential in order to complete work within the allotted time frame, as staff may require help from team members. Collaborative experiments will be more common. For example, researchers in Pod A could process blood samples, isolate, and prepare cells during the first shift, and researchers in Pod B could carry out functional assays on these cells during the second shift.
- Experimental planning: all researchers recognize that time management is a key skill that must be employed to efficiently plan a working day. However, as mentioned above, staff will need to adhere to strict timelines. Therefore, time management and experiment planning will be the key to efficient working. Experimental plans must be made and agreed in advance to allow for rota organization.

### Equipment Usage

In order to ensure a safe working environment using shared equipment, clear rules must be put in place as follows:

- Equipment booking systems: to ensure social distancing is maintained, equipment usage should be booked in advance. If online systems are not available for this, a simple shared calendar will meet this need. Only one person should use equipment at any one time and social distance measures must be put in place around the equipment area. Extra time must be added in for each session to allow for equipment cleaning, plus an extra 15 minutes [33] to allow any aerosols to settle before the next person enters the space.
- Removal of nonessential shared equipment: any nonessential equipment such as coffee machines or microwaves in communal areas should be removed.
- Equipment cleaning: all equipment should be wiped down with 70% ethanol before and after use. Cleaning of equipment should be documented to ensure that it is done and to indicate to the next person that the area is clean.
- Equipment maintenance: procedures must be put in place for equipment repair or maintenance. Engineers must only be allowed on site to attend to equipment if it is safe for them to do so. Such individuals must not come into contact with any staff members unless it is absolutely necessary.

### PPE and Hand Gel Stations (Staff Hygiene)

In order to limit the risk of transmission, clear rules and signage must be put in place as follows:

- Appropriate PPE as stated in the local risk assessment should always be worn. In the event that social distancing is not possible, masks and goggles may be required. In these instances, a risk assessment should be written and approved and appropriate PPE identified.
- Personal PPE such as lab coats and goggles should be stored separately to prevent cross contamination.
- It may be beneficial to provide hand sanitizers around the workplace. This will encourage staff members to keep their hands clean and therefore help to reduce fomite transmission.

### Amended Standard Operating Procedures and Risk Assessments

In order to ensure sample types are handled safely, changes to operating procedures must be made as follows:

- All local risk assessments and standard operating procedures should be reviewed and, if required, amended and reapproved by local committees. All clinical samples should be assessed as recommended in the previous section.
- It is important that samples that are COVID-19 confirmed or of unknown COVID-19 status are stored and labeled appropriately.
- Designated sample reception areas are needed. In order to ensure safety, designated sample reception areas should be established for samples that are COVID-19 positive or those with an unknown COVID-19 status. Such areas should be clearly marked. Appropriate waste receptacles, PPE donning and doffing areas, and disinfectants should be available at these stations. Individuals working in such areas must wear the appropriate PPE as stated in the documentation and the location of the reception area must be selected with social distance measures in mind.

### Work Checks

It is important to have a record of which staff members are on site at any given time. This information will be required if an outbreak does occur in the workplace in order to carry out contact tracing and to ensure all the appropriate staff self-isolate if required. It is important to ensure that all staff members adhere to the local rules laid out with appropriate checks conducted to emphasize best practices.

## Discussion

These proposed flowcharts and working patterns are suggested to identify and mitigate risk to research participants and staff during the COVID-19 pandemic. The central components of these guidelines are based on advice from Public Health England and the UK government but can be adapted as needed.

## Appendix

Multimedia Appendix 1 Risk assessment.

## Abbreviations

Cat2+: enhanced category 2
Ct: cycle threshold
HG3: Hazard Group 3
PCR: polymerase chain reaction
PPE: personal protective equipment
RNA: ribonucleic acid
