# Supplementary material for: Mitigating Health Risks to Reopen a Clinical Research Laboratory During the COVID-19 Pandemic: A Framework
Source: JMIR Res Protoc. 2020 Dec 4;9(12):e22570. doi: 10.2196/22570 (PMC7721628; doi:10.2196/22570)
Supplement: Multimedia Appendix 1 [file resprot_v9i12e22570_app1.pdf]

**For Safety Services use only.** ASSESSMENT NO.

RISK ASSESSMENT of WORK with BIOLOGICAL MATERIALS

PLEASE READ:

Health and Safety Policy requires that a risk assessment of all work with biological materials must be carried out in advance of work commencing. Any work involving:

1. **higher risk categories (ACDP 2 or above);**
2. **animal or plant pathogens requiring a license from DEFRA/Animal and Plant Health Agency/HSE;**
3. **other categories of work or activities notified to Budget Centres by the Advisory Group on the Control of Biological Hazards**

must be notified to and approved by the Advisory Group on the Control of Biological Hazards unless a specific exemption applies.

Assessments for Hazard Group 1 agents or other work that falls into Containment Level 1 should be scrutinised by the local Biological Safety Officer. The BSO should contact Safety Services for advice if there are any doubts over the classification of the work.

YOU SHOULD COMPLETE ALL OF PART A, THE APPROPRIATE SECTION(S) OF PART B, AND ALL OF PART C. THE FORM SHOULD BE SUBMITTED TO SAFETY SERVICES FOR REVIEW VIA YOUR BIOLOGICAL SAFETY OFFICER.

**Date submitted:**		**Date approved:**	

PART A: Please provide the following general information:

**School/Department:**	

**Principal investigator:**		**Position:**	
**E-mail address:**		**Phone no.:**	
**Author of assessment**		**Email:**	

Please give a brief and descriptive title for this risk assessment

Title:	Project A<br>Project B<br>Project C

Aims and objectives of investigation: What are you doing, and why?
For aims, objectives and techniques for the individual projects stated above, please refer to table in Appendix 1<br><br>COVID-19 precautions<br><br>For all projects listed above, the aim is to collect whole blood samples from patients of unknown SARS-CoV-2 status (not actively exhibiting any symptoms specific to COVID-19 but with no rtPCR confirmation of a negative viral load). For projects A and B sputum are also required respectively. However, due to the high risk of these samples (see information below), sputum and saliva samples will not be collected for these projects until it is deemed safe to do so.<br>Patients will undergo a pre-screen telephone appointment with a Research Nurse. This will be presented as a questionnaire which will be standardised for all patients enrolled on the study Appendix 2. On arrival at the clinic, individuals will be screened prior to providing a sample by a Clinical Research Fellow or a Research Nurse who will undertake a clinical assessment of the patient to determine whether the patient is exhibiting any characteristic symptoms (a new continuous cough, a high temperature (37.8 or above) and a loss of, or change in normal sense of smell or taste) of COVID-19 (See workflow (Appendix 2) for more details). Assessment will take into consideration any medical conditions that the patient may already have (see Appendix 1) i.e. COPD and AATD. No samples will be taken from patients presenting with symptoms characteristic of COVID-19.<br><br>Whole blood will be the only sample taken for these studies (as indicated above, respiratory samples will not be taken until it is safe to do so). Studies have shown that the likelihood of virus being detected in whole blood from COVID19 positive patient samples is low (see more details further down this document). Although both the sample and study participants are deemed low risk (no clinical COVID-19), samples will be processed at category 2 containment level but with additional aerosol free procedures in place as indicated below and in the SOP (Appendix 4) to take into consideration that study participants may be asymptomatic carriers of SARS-CoV-2.

Workers initially involved in work:	Post/experience/training:
(name) (Study name)<br><br>Dr (name) (Study name)<br><br>Dr (name) (Study name)	(name) is a highly trained, competent and experienced final year PhD student with extensive experience in the techniques required for this study. 3 years laboratory experience.<br><br>(name) is a Clinician who is highly trained, experienced and fully competent in the techniques required for this project. Over 3 years laboratory experience.<br><br>(name) is a Research Technician with over 13 years laboratory experience including category 2 and 3 work. She is highly trained and experienced in all the techniques required for this study

Training and Assessment of Competence

Specify arrangements for provision for existing and future personnel

All staff will be fully trained and competent at working in a class II microbiological safety cabinet at containment level 2 and with human blood and blood products. All staff will be very familiar with all the techniques required, will have read and understood the standard operating procedures and risk assessments and will be aware of the potential risks involved and will be fully competent in safety critical procedures and the use of equipment. All training will be documented and signed off by both the trainer and trainee.

All Researchers attend College Health and Safety Training.

(department name) Lab Manager (name) will carry out initial lab induction to inform about potential risks and waste streams. Researchers are supervised by experienced researchers (at least six months experience) until the competent researcher deems the trainee to be competent.

Additional aerosol free workflow training will be provided by (name) (position), (name) (position) and (name) (position)

Principal areas where work will be carried out:	
**Room No. and location**	**ACDP Categorisation – containment level?**
Laboratory (name or number), (CL2) situated (name of institute, department and location ID)<br><br>Samples reception will take place in a designated area of Laboratory (name or number). This will be clearly signed.<br><br>All blood processing will take place within Class II MSCs which are located in an inner room within Laboratory (name and number).	CL2

****

PART B: Please provide information in one or more of the following sections, as appropriate. Only the sections that you complete should be submitted.

Section 1: *micro-organisms (prions, viruses, bacteria, fungi, parasites in ACDP categories 2 - 4 and pathogens controlled by the Department for the Environment, Food and Rural Affairs). Work with ACDP category 4 pathogens is not currently permitted in the University.*
Section 2: *human tissues and body fluids*
Section 3: *cell cultures*
Section 4: *plants and plant material*
Section 5: *animals and animal tissues*

Section 1: Microorganisms note:

1a. Nature of Microorganism

Name and Strain - Does it cause disease in humans, animals or plants?	ACDP Haz Gp*	Source	Susceptibility/ resistance to clinically useful antibiotics or other treatments	Is this a virulent or attenuated strain?
**1b. Has any strain been genetically modified in any way? Yes / No (Delete, as appropriate)**				
**If yes, complete GM *pro forma***				

*See the Approved List of Biological Agents for human pathogens (produced by Advisory Committee on Dangerous Pathogens). See guidance from Defra/Animal and Plant Pathogen Agency when handling animal or plant pathogens.

2. Risk to human health from microorganism

**Describe effects on human health *(including colonisation, infection, allergy, toxin-mediated disease)*:**
**Humans at increased risk of infection etc.**<br>***(including immunocompromised, pregnant women, breast-feeding mothers)*:**

3. Quantity of organisms to be used

volumes to be worked with	concentrations of organisms to be used

4. Environmental Considerations (Risk to animals, fish, plants etc).

5. Using the above information, what Containment Level is appropriate?

ACDP level:	

Section 2. Human tissues and body fluids

1. Nature of tissues and body fluids

**Site of tissue, nature of body fluid(s):**
This risk assessment covers blood samples taken from patients with an unknown COVID19 status. This is detailed in Appendix 1.<br>It has been assessed that blood samples are low risk for the reasons stated below;<br><br>Participants do not have clinical COVID19<br>Participants donating blood will be pre-screened by research nurses before arrival using the questionnaire in Appendix 3. On arrival, patients will be clinically assessed as specified previously in this document and will only be sampled if they do not exhibit any symptoms characteristic of COVID19 (see Appendix 3 for assessment questionnaire for more details). However, there is a risk that they may be asymptomatic.<br><br>Risk of blood products containing infectious virus is low<br>SARS-CoV-2 virus has been detected in the blood of infected patients. However, to date, research has indicated that only a small number of infected patients have detectable viral RNA in the blood and that levels of detection are low compared to respiratory samples (BAL, sputum and nasal and pharyngeal swabs). RNA detection in the blood has varied from 0% (Wolfel et al), 1% (Wang et al), 8% (Young et al), 11.5% (Chan et al) to 15% (Huang et al) of patients. In addition to this, there is as yet, no evidence of any type of coronavirus being transmitted through blood donation (https://www.blood.co.uk).
All processing will be carried out in a MSC class II. Samples will be put into secondary containment during incubation periods and will be fixed as indicated below prior to being analysed by flow cytometry and light microscopy.<br>Inactivation methods<br>4% Paraformaldehyde<br>Neutrophils will be fixed in 4% PFA (Darnell et al) prior to FACS analysis.<br>100% Methanol<br>Coronaviruses are highly sensitive to alcohol, which disrupts the outer membrane. A 30 second exposure to 70-80% Ethanol has been shown to inactivate coronaviruses to below the limit of detection (>4 log reduction Rabenau H et al). Neutrophils will be fixed in 100% Methanol prior to light microscopy.<br><br>For references See Appendix 5

2. Details of human subjects from whom tissue/body fluid is obtained (including likely presence of infective agents, e.g. blood borne viruses):

Please refer to table in Appendix 1 for information on where patients will be recruited from for each project stated in this risk assessment and what type of sample will be taken.

Patients will not be screened for SARS-CoV2 or blood borne viruses (BBVs) unless displaying clinical symptoms as judged by respiratory consultant (see Appendix 3 for assessment questionnaire).
Any patients / samples with COVID19/SARS-CoV-2 or known BBVs such as HIV or HepB will be excluded from the study and samples will not be processed.

Although samples from the above patient cohorts are deemed low risk for the reasons stated above, all samples will be processed in a class II containment cabinet using aerosol free workflows to minimise risk from potential asymptomatic carriers of SARS-Co-V-2.

3. Infective Risk

**Likelihood of infective risks to laboratory workers:**
The protocols described in the respiratory laboratory SOP (Appendix 4) for this project ensure that the infective risk to laboratory workers is very low.<br><br>Samples known to contain BBVs such as Hep B and HIV will not be used. Samples will not be taken from patients with a known COVID19 status or if patients present with COVID19 symptoms as described previously in this document.<br><br>This will be further reduced by elevated safety measures and aerosol free working.<br><br>All laboratory staff named above will be trained in operating under aerosol free conditions and in proper use of PPE by laboratory managers before sample collection.

4. Interim Containment in light of information above

**ACDP level:**	MSC II in CL2.

Ethical Approval: PLEASE NOTE

Work with human tissue/body fluids usually requires ethical approval and consent in line with the Human Tissue Act and current policy. Does this project have current approval by a local Ethics Committee? Has consent been obtained from the donor for the use of the blood/tissue (where required)?

Ethical approval has been received for all work stated for all projects.

(study name) (Ethics codes)

Section 3: Cell cultures

1. Nature of cells

Name	Low/Medium/High Risk?	Species and anatomical origin
(Cell type) (name of researcher using the cells) (researchers project title	i.e low	i.e Blood

2a: Source of cells

Are the cells derived from a person who currently has access to the laboratory where the work will be performed?

yes

NB Persons MUST NOT work on their own cells

**If yes, what precautions are to be taken to prevent that person being exposed to their own cells?**
No individual will process or culture their own cells.

2b: Are cells modified?

**Have they been modified in any way (eg, by transfection, transformation etc.)?**
**If yes, complete GM *pro forma* also**

3. Risk to humans

**What are the likely effects on human health (including risk of infection, e.g., presence of blood-borne viruses, cell products):**
Risk from blood borne viruses (BBVs)<br>Human whole blood and blood products may potentially contain blood borne viruses (BBVs) or other agents harmful to health so carry a risk of infection if not handled correctly. Such samples will therefore be handled at category 2 in a containment level 2 cabinet and users handling such samples will always wear a lab coat and gloves.<br><br>COVID19 risk<br>Current evidence as detailed previously in this document suggests that blood is an unlikely route of infection for the SARS-CoV-2 virus. This risk assessment will be updated if any further evidence on potential infectious risks from blood products becomes apparent. But until such evidence presents, any samples with an unknown COVID19 status will be handled in a class II containment cabinet under category 2 conditions using aerosol free work processes as detailed in the SOP (Appendix 4).<br><br>Samples known to be SARS-CoV-2 positive or that contain BBVs such as Hep B and HIV will not be used.
**Humans at increased risk of infection etc.<br>(including immunocompromised, pregnant women, breast-feeding mothers):**
Staff who are known to be Immunocompromised will not work on blood samples unless they have been inactivated by the methods stated previously in this document.

4. Environmental Considerations

Samples will be inactivated in an appropriate disinfectant or autoclaved as indicated in the laboratory SOP (Appendix 4). No harm to the environment.

5. Are animal/plant pathogens likely to be present?

**Is use of cells likely to require HSE or DEFRA approval because of nature of any endogenous pathogens?**<br>**/ No (delete as appropriate)**<br>**Please expand on the nature of these pathogens (e.g. are they Specified Animal Pathogens etc):**<br><br>No HSE or DEFRA approval required.
**If yes, approval will not be granted until an appropriate licence has been obtained**

6. Interim Containment, taking into account information above

**ACDP containment level**	N/A

PART C CONTROL MEASURES

1. Are any of the work procedures likely to generate aerosols, either deliberately or accidentally? If so, should the work be undertaken in a Microbiological Safety Cabinet or be contained in some other way?

All human blood and blood products will be routinely handled at containment level 2 in a class II biological safety cabinet with additional aerosol tight procedures to prevent exposure to adventitious pathogens that may be present in the samples. A lab coat and two pairs of disposable nitrile gloves will be worn at all times.

Procedures likely to generate aerosols

Pipetting

Pipetting is a possible aerosol generating procedure, as such this must always be done inside a class II safety cabinet. Fluid must be ejected from the pipette carefully and pipette tip must be in contact with the wall of the tube and sample released slowly.

Flow cytometry

Flow cytometry will generate aerosols. The flow cytometer is not located within a MSC. Therefore, all samples will be fixed in PFA which will inactivate the virus if present (see information documented previously in this RA on methods of fixation) Prior to being transferred to the flow cytometer.

Accidental aerosol generation

Accidental aerosol generation may occur if a tube breaks in the centrifuge or if a tube is dropped. A tube dropped inside a cabinet will be contained and will be dealt with as indicated in the spillage procedures section below. All tubes will be immediately capped and transferred to secondary containment prior to being removed from the MSC. In the case of centrifugation, tubes will be placed in centrifuge pods within the MSC and the pods sealed prior to removal from the cabinet. Therefore, a spillage outside the hood is very unlikely. However, we have outlined clear protocols for cleaning up a spillage in further down in this document. The largest volume of liquid in a single tube will be 15ml during the process of neutrophil isolation and in this case the blood will have already have been diluted and the neutrophils purified and washed. This process will be carried out over an absorbent mat and within a MSC. We therefore believe that the risk will be low. There are also clear processes outlined further along in this document regarding the use of centrifuge and what to do in the event of a breakage.

Sample transport

All samples must be transported in a secondary containment vessel. Only sealed vessels to be admitted to the laboratory. Samples will be received in the designated sample reception area in laboratory (name). This will be clearly signed and used only for sample reception.

The secondary container will be decontaminated with 70% Ethanol in the sample reception area. Secondary containment will only be unsealed after transfer to MSCII hoods for processing.

Sample processing

All sample transfers, manipulation and processing will occur within MSCII hoods. Operators will wear a minimum of 2 pairs of nitrile gloves at all times when handling open samples. One pair will be discarded when withdrawing, which will remain in the hood until experiment end when it will be disposed of into a disposafe jar. This process will be repeated every time hands are withdrawn until final decontamination at experiment end. Samples requiring further external processing e.g. flow cytometry will be sealed until immediately prior to use. Where required (i.e. any process that is likely to generate an aerosol outside of a hood), samples will be fixed in a validated fixative indicated previously in this document thus further reducing risk.

Possible aerosol generating procedures during sample processing (luminex, flow cytometry) should use automated loading procedures where appropriate. In such cases users should ensure local laboratory users are aware and maintaining appropriate distancing measures.

Sample centrifugation
Samples will be centrifuged in sealed buckets only, Buckets will be transferred into the hood, loaded and sealed. Centrifuge pods should be wiped with 70% Ethanol prior to removal from the hood. Unloading of samples after centrifugation will also occur within the MSCII containment hood.

Vortexing of samples
Samples of unknown COVID-19 status will never be vortexed uncapped. Where possible samples will be vortexed within a class II cabinet. Where this is not possible and it is deemed necessary to mix samples, samples must have appropriate sealable, screw top lids and be placed in a sealable plastic bag (secondary containment) prior to vortexing. 70 % Ethanol must be sprayed in the bag prior to removing the tube.

Lab coats
As possible routes of viral transfer all lab coats will be stored independently to ensure no physical transfer. Labs coats should be decontaminated with 70% Ethanol at end of experimental procedure. Storage facility will be provided for lab coats not in use to minimise risk of transfer.

Decontamination
MSCII cabinets will be fully decontaminated at experiment end with Chemgene (1%)/Ethanol (70%) to ensure no chain of contamination.

2. Will the work require the use of sharps? If yes, is there a safer alternative?

Use of sharps while taking blood samples
Blood will be taken in a designated area (phlebotomy room) by NHS professionals trained in phlebotomy so there is no risk to laboratory workers form sharps. Volunteers will undergo a clinical assessment prior to providing a sample. This process is outlined in the flow diagram in Appendix 2. Blood will not be taken from individuals presenting with COVID19 symptoms.

A lab coat, gloves and facemask will be worn at all times while taking blood samples. Sharps are disposed of in yellow sharps bins immediately and disposed of in accordance with the (name of institute) waste stream.

Use of sharps during sample processing
Sharps present a risk of infection to laboratory staff handling whole blood. Therefore, unless deemed essential to the protocol, use of sharps should be prohibited.
This protocol requires use of glass slides to perform a differential cell count and this is a necessary part of the procedure so can therefore not be avoided as there is currently no alternative. However, risk of cuts and abrasions from glass slides while handling blood for this protocol is very low, as glass slides are inserted into cassettes outside of the hood. Whole blood will undergo processing to produce purified cell suspension which will then be added to the cassette containing the slide inside the MSC. Therefore, all other blood components will have been removed and disposed of and the isolated cells will have been washed and diluted to $1x10^6$ cells per ml. As such, the risk of infection from the glass slide will be very low. All staff will be aware of the risk of glass slides. A lab coat and gloves will be worn at all times when handling unfixed slides. In the event of a cut or abrasion from a glass slide, gloves should be removed and the area washed in soapy water immediately. Medical advice should be sort and the laboratory manager informed. The incident should be recorded.
No other sharps will be used.

3. Protective equipment and clothing to be used

Use of a microbiological safety cabinet (MSC)
A class II biological safety cabinet will be used for processing of human blood and blood products.

Lab coats
A lab coat will be worn at all times when processing human blood. The lab coat must be fully fastened. Lab coats will not be shared. Each staff member will have their own lab coat. When not in use, lab coats will be stored in individual plastic bags which will be hung on individual staff members hooks in the laboratory (location, laboratory 2 (institute name) research laboratories) to ensure no physical transfer.
Labs coats will be decontaminated with 70% Ethanol at end of experimental procedure prior to being stored in a bag.

Gloves
Disposable nitrile gloves will be worn at all times when working with human blood and blood products. Two pairs of gloves will be worn while working in the MSC and staff members will be trained in safe donning and docking procedures to prevent contamination of the inner gloves or hands.

4. Storage arrangements

Human blood processed in Research Labs will be stored in secondary containment at -80ºC or -20ºC until analysed. Samples taken during the period of the COVID19 pandemic will be clearly labelled to identify them and to prevent such samples being opened out of a containment cabinet in the future. A record of samples will be kept.

5. Transport:

**Are materials to be moved outside the laboratory suite *(e.g., between labs or buildings).* If so, please outline how this is to be done.** **If materials are to be sent by air/road/rail please outline the packaging requirements (UN 2814/UN3373/ UN 3245 or other) and other arrangements for shipping.**
Human blood and blood products taken from consented patients or volunteers will be transported to the (Institute name) in secondary containers, including approved transport bags suitable for Cat. B containment. Samples will be received in a designated area in laboratory 2 and removed from tertiary containment for decontamination with 70% Ethanol. Secondary containment will not be unsealed in general lab area, unsealing only after transfer to MSCII cabinet I the inner room of laboratory (name) for unsealing and processing.<br>Human materials that leave the labs will be transported in approved transport bags suitable for Cat. B containment or in packaging that conforms to UN3373.<br>All transport containers and viles will be decontaminated with 70% Ethanol after use.

6. Disinfection

**Specify concentration of disinfectants and minimum time of treatment. Have these disinfectants been validated for use with the recipient microorganism?**
Coronaviruses are highly sensitive to alcohol, which disrupts the outer membrane. 70% Ethanol has been approved for used against the virus (as indicated previously in this document).<br><br><u>Spillages</u><br>Any spillages to be swabbed with fresh1% Virkon which will be prepared daily or covered with preset granules. Spillage procedures are specified in further on in this document.<br><br><u>MSC cleaning</u><br>10% Microsol or 70% Ethanol will be used to decontaminate safety cabinets before and after sample processing. Manufacturer's data shows Virkon and Microsol to be effective against BBVs at this concentration.<br><br><u>Liquid waste</u><br>Liquid waste will be collected in a suitable receptacle (discard pot) which will then be sealed, sprayed with 70% Ethanol prior to removal from the MSC. Liquid waste pots will be autoclaved then the liquid discarded down a designated sink.<br><br><u>Waste in the MSC</u><br>Disposable outer gloves, pipette tips and tissues used for wiping the cabinet will be disposed of in a disposafe jar within the cabinet. This will be sealed and sprayed with 70% Ethanol prior to removal from the cabinet. It will be placed directly into an autoclave bag, sealed with autoclave tape and autoclaved. It will then be disposed of by the usual (institute name) waste stream.<br><br><u>Equipment</u><br>Centrifuges and other equipment will be wiped down with 70% Ethanol after use.

7. Sterilisation and disposal procedures

Plastic consumables
All contaminated plastic consumables such as centrifuge tubes will be autoclaved (135°C 15 mins under pressure) before removal as clinical waste by licensed contractors using the (institute or hospital name) waste stream.
Pipette tips will be ejected immediately into disposafe jars, contained within MSC Class 2, these will be autoclaved to 135°C 15 mins under pressure and disposed of via the (institute or hospital name) waste stream.

Paper waste
Contaminated paper tissue to be placed in yellow clinical waste sacks.

Blood tubes
Empty blood tubes (containing blood residue) to be placed in yellow topped bin and disposed as clinical waste in accordance with the (institute or hospital name) waste stream.

Liquid waste
Blood over 50ml will be disinfected with 1% Virkon, by adding 1 tablet or one scoop of powder per 100ml of liquid and set using Vernagel. This will be done inside a MSC. This will then be disposed of in a yellow clinical waste sack in accordance with the (institute or hospital name) waste stream.

Glass slides
Glass slides are fixed in methanol and may be stored for further reference or disposed of at bench/ location they are used, in yellow sharps bins. These are disposed of in accordance with the (institute or hospital name) waste stream.

MSC
MSCII cabinets will be fully decontaminated at experiment end with chemgene (1%)/Ethanol (70%) to ensure no chain of contamination.

8. Accident and Emergency procedures

**e.g. In the event of spills or accidental exposure to microorganisms (specify disinfectants and any special containment for large volumes); Loss of containment; Escape of an animal, etc. Please note that certain categories of incident/accident are reportable to the Health and Safety Executive.**
Emergency spillage procedures<br>All spillages should be dealt with immediately and reported to the laboratory manager.<br>Spillage within the MSC<br>All work within a safety cabinet should be carried out over an absorbent mat. In the event of a spillage inside the containment cabinet, the matt should be sprayed with 70% Ethanol then disposed of in the Disposafe jar. A new mat or absorbent tissue should be used to soak up the rest of the spillage and the area should be thoroughly decontaminated with 70% Ethanol. Outer gloves must be changed prior to continuing work.<br><br>Spill outside the MSC<br>In the event of a spillage outside the cabinet, absorbent material should be used to contain and soak up the spillage and a fresh solution of 1% Virkon poured over, or for larger spills covered with presept granules. This should be left for the validated contact time (2 minutes on hard surfaces). Waste will be placed in an autoclave bag, autoclaved then disposed of in yellow clinical waste bags. The contaminated area should undergo a further clean with 70% Ethanol.<br><br>Contamination of individuals<br>In the event of the contamination of an individual with a positive or suspected COVID-19 positive sample, contaminated PPE or clothing should be removed immediately and carefully and disposed of directly into an autoclave bag and sealed. If area of contamination is small, 70% Ethanol can be sprayed on the contaminated area prior to removal. Hands must be washed thoroughly once gloves are removed. Individual should exit the lab and immediately inform laboratory manager who will assess any further action required. All other users of the laboratory should be notified to prevent them from entering the laboratory until it is deemed safe to do so. In the event of a large spillage see emergency spillage procedures.<br>In the event of contamination of skin, area must be washed thoroughly with soapy water and further advice must be sort.<br>All individuals possible exposed to COVID19 must report the incident to the enforcing authority and immediately seek advice from a medical professional in regards to post-exposure treatment. Any individual who has had a potential exposure to COVID-19 must self-isolate until they have had a negative PCR test.<br><br>Breakage of tubes within the centrifuge:<br>In the event of a suspected accident during centrifugation such as excessive noise and vibration, immediately turn off the centrifuge. Do not open the lid of the centrifuge for at least 30 minutes after the rotor has come to rest, to allow time for aerosols to settle-however use of sealed buckets should significantly minimise this risk. Post prominent warning notices to stop others from opening the centrifuge. Remove debris from centrifuge and decontaminate with 10% Microsol.<br><br>**All accidents and incidents should be promptly reported and documented**

9. Occupational Health issues/Health Surveillance required

**Consider the need for immunisation, chemoprophylaxis, health monitoring, arrangements for record keeping of exposure to ACDP category 3 organisms; Also, health surveillance (e.g. if handling animals); allergy reporting if handling plant material, etc**
All staff processing human blood are advised to have up to date HepB immunisations. Those not wishing to have the immunisation require authorisation from their PI stating that '**the biological risk from the samples is acceptable without immunisation'.**

10. Are any other control measures required?

None

11. What is the final Containment Level for this work?

CL2

THIS FORM SHOULD NOW BE EMAILED TO YOUR BIOLOGICAL SAFETY OFFICER FOR INTERNAL APPROVAL. YOUR BSO WILL THEN FORWARD THIS FORM TO SAFETY SERVICES FOR REVIEW AND APPROVAL BY THE ADVISORY GROUP.

Appendix

Appendix 1: Table: Project information

Insert project table here

Appendix 2: Flow diagram of assessing patient and sample risk

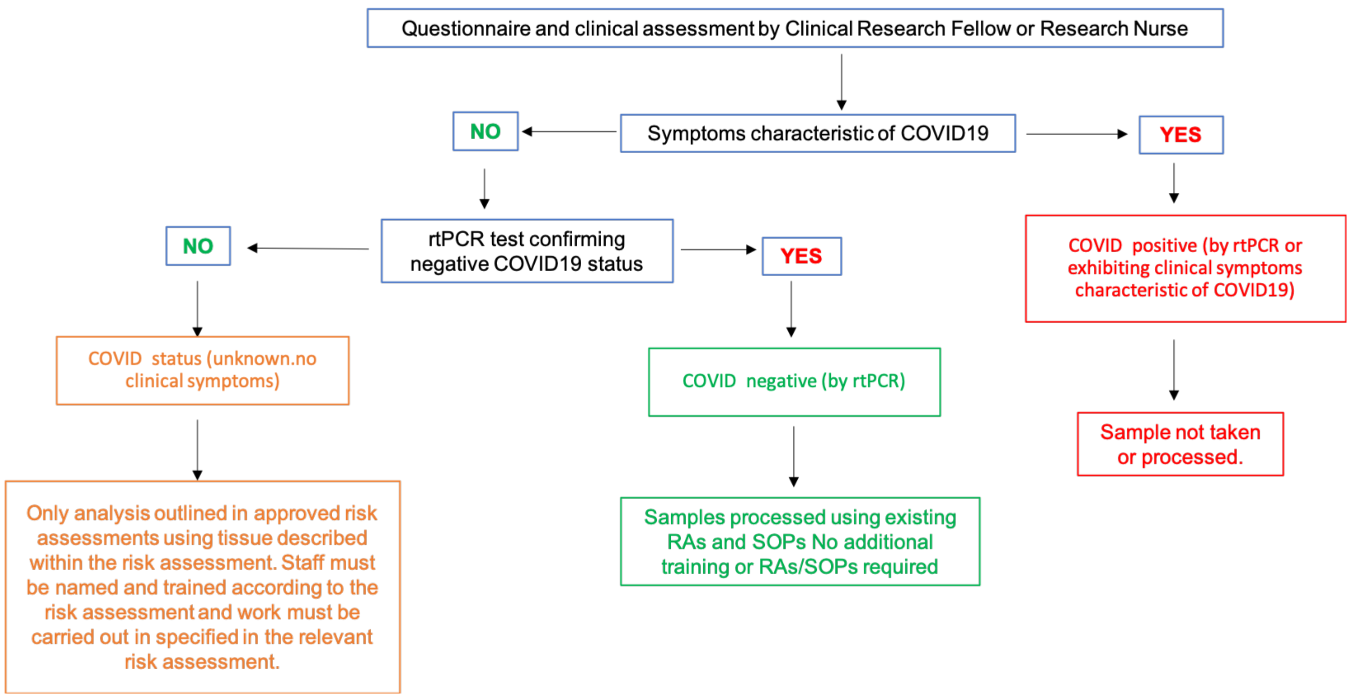

Appendix 3: Patient assessment questionnaire

COVID-19 pre-visit Screening Questionnaire

Each patient offered an appointment will receive a phone call, 24 hours before their appointment, from a team member to complete this questionnaire:

1. Does the patient or member of their household have symptom(s) suggestive of COVID 19?

   Yes/No

   If yes, appointment cannot proceed until 14 days have elapsed & patient remains on a pending list.

   Covid-19 symptoms:

   - a new continuous cough.
   - a high temperature (37.8 or above).
   - a loss of, or change in, your normal sense of taste or smell (anosmia).

2. Is the patient or member of their household awaiting result of a Covid-19 swab ?

   Yes/No

   If yes, appointment cannot proceed until a confirmed negative Covid-19 swab result.

   If Covid-19 swab result is positive, appointment cannot proceed until 14 days have elapsed

3. Has the patient or a member of their household been contacted by NHS Test and Trace within the last 14 days to advise to have a Covid-19 swab and/or self-isolate?

   Yes/No

   If yes, appointment cannot proceed until 14 days have elapsed and patient has completed testing and/or self-isolation period advised by NHS Test and Trace.

4. Is the patient ‘shielding’ due to immunosuppression or other health risk?

   Yes/No

   If yes, appointment should be arranged to proceed in accordance with the latest PHE guidance. Might involve the use of a designated ‘cold area’, but local guidelines should be followed.

5. We want to understand how many close contacts the patient has had apart from members of their household in the last 48 hours.

Close contact means:

a. Patient having face-to-face contact with someone (less than 1 metre away)
   Yes/No
b. Patient spending more than 15 minutes within 2 metres of someone
   Yes/No
c. Patient travelling in a car or other small vehicle with someone (even on a short journey) or close to them on a plane.
   Yes/No
d. Patient work in – or have visited – a setting with other people **where strict social distancing was not followed** (for example, a GP surgery, a school or a workplace)
   Yes/No

If answers to any of the above are "Yes", then defer appointment for 14 days

6. Inform patient of the next sequence of events.

   Risk:benefit analysis: Patients should be warned that there is a very small increased risk of contracting COVID-19 inherent in attending the department.

   Inform patient that there is a higher than usual chance of last minute cancellation due to current unpredictable circumstances re PPE, staffing, etc.

Appendix 4: Respiratory Laboratory SOP

Insert your laboratory specific SOP here. This may need to be amended and approved by the appropriate local health and safety authority.

Appendix 5: References

References

Wölfel R, Corman VM, Guggemos W, et al. Virological assessment of hospitalized patients with COVID-2019. Nature. 2020. Epub ahead of print.
Wang W, Xu Y, Gao R, et al. et al. Detection of SARS-CoV-2 in Different Types of Clinical Specimens. JAMA; 2020. Epub ahead of print.
Young BE, Ong SWX, Kalimuddin S, et al. Epidemiologic Features and Clinical Course of Patients Infected With SARS-CoV-2 in Singapore. JAMA; 2020. Epub ahead of print.
Chan JF, Yip CC, To KK, et al. Improved molecular diagnosis of COVID-19 by the novel, highly sensitive and specific COVID-19-RdRp/Hel real-time reverse transcriptionpolymerase chain reaction assay validated in vitro and with clinical specimens. J Clin
Micro; 2020. Epub ahead of print.
Huang C, Wang Y, Li X, et al. Clinical features of patients infected with 2019 novel coronavirus in Wuhan, China. Lancet; 2020. 395:497-506.
Darnell, M. et al Inactivation of the coronavirus that induces severe acute respiratory syndrome, SARS-CoV Journal of Virological Methods (2004) 121: 85-91
doi:10.1016/j.jviromet.2004.06.006
